# The Morphogenesis of Cranial Sutures in Zebrafish

**DOI:** 10.1371/journal.pone.0165775

**Published:** 2016-11-09

**Authors:** Jolanta M. Topczewska, Ramy A. Shoela, Joanna P. Tomaszewski, Rupa B. Mirmira, Arun K. Gosain

**Affiliations:** Division of Pediatric Plastic Surgery, Stanley Manne Children’s Research Institute, Northwestern University Feinberg School of Medicine, Ann & Robert H. Lurie Children's Hospital of Chicago, Chicago, Illinois, United States of America; University of Sheffield, UNITED KINGDOM

## Abstract

Using morphological, histological, and TEM analyses of the cranium, we provide a detailed description of bone and suture growth in zebrafish. Based on expression patterns and localization, we identified osteoblasts at different degrees of maturation. Our data confirm that, unlike in humans, zebrafish cranial sutures maintain lifelong patency to sustain skull growth. The cranial vault develops in a coordinated manner resulting in a structure that protects the brain. The zebrafish cranial roof parallels that of higher vertebrates and contains five major bones: one pair of frontal bones, one pair of parietal bones, and the supraoccipital bone. Parietal and frontal bones are formed by intramembranous ossification within a layer of mesenchyme positioned between the dermal mesenchyme and meninges surrounding the brain. The supraoccipital bone has an endochondral origin. Cranial bones are separated by connective tissue with a distinctive architecture of osteogenic cells and collagen fibrils. Here we show RNA *in situ* hybridization for *col1a1a*, *col2a1a*, *col10a1*, *bglap/osteocalcin*, *fgfr1a*, *fgfr1b*, *fgfr2*, *fgfr3*, *foxq1*, *twist2*, *twist3*, *runx2a*, *runx2b*, *sp7/osterix*, and *spp1/ osteopontin*, indicating that the expression of genes involved in suture development in mammals is preserved in zebrafish. We also present methods for examining the cranium and its sutures, which permit the study of the mechanisms involved in suture patency as well as their pathological obliteration. The model we develop has implications for the study of human disorders, including craniosynostosis, which affects 1 in 2,500 live births.

## Introduction

The objective of this study is to further understand the zebrafish cranial vault and suture development and their homeostasis in adult animals. This topic has only briefly been described in previous work [1, 2].

The vertebrate head skeleton is a complex structure composed of the neurocranium (skull base and vault) and viscerocranium (jaw and branchial arch derivatives), which originate from the neural crest (NC) and mesodermal mesenchyme [3–7]. The skull vault, or roof of the neurocranium, serves to protect the brain while concurrently permitting skull expansion during development. It is composed of two types of bilaterally developing bones: frontal and parietal. These bones are formed in the process of intramembranous ossification, in which condensing mesenchymal cells directly differentiate into the osteogenic linage. The fifth bone of the cranial vault, the supraoccipital bone, is of endochondral origin and requires an intermediate cartilage template. All cranial bones are formed between dermal mesenchyme and the meninges surrounding the brain [3]. Fate mapping experiments have revealed that the mouse frontal bone originates from NC lineage while the parietal bone develops from the mesoderm [6, 8]. The zebrafish frontal bone has dual origin; its anterior part arises from the NC and its posterior from the mesoderm. The cartilaginous epiphyseal bar underneath the frontal bones demarcates the border between the anterior and posterior segments [5]. The parietal bone originates from the mesoderm in both zebrafish and mice. Cell lineage-tracing studies in mice suggest that calvarial bones develop independently from the dermal mesenchyme, therefore the term “membrane bones”, as opposed to the term “dermal bones”, has been proposed as being more adequate [4].

Fibrous joints, called sutures or syndesmoses, separate the opposing edges of the cranial bones [1, 9]. Sutures are identified with respect to their adjacent bones, i.e., the interfrontal suture (metopic in humans) lies in between the frontal bones. The coronal suture separates the frontal and parietal bones, the sagittal suture separate the parietal bones, and the lambdoid sutures separate the occipital and parietal bones [10]. Because mutations in genes encoding FGF receptors and transcription factors like Twist1 and Msx2 cause premature suture obliteration and fusion of cranial bones, it is accepted that sutures serve both as growth and signaling centers [11–15].

In mammals, the cranial sutures have an important role in skull functionality as they are maintained patent long enough to sustain growth of cranial bones while being coordinated with the overall growth of the neurocranium. Delayed ossification of the cranium results in wide-open fontanels and suture agenesis, while the accelerated growth of cranial bones causes suture obliteration and craniosynostosis disorders in humans [16]. Premature fusion of cranial bones in humans occurs in approximately 1 in 2,500 live births [17, 18]. This restricts the growing brain, which can lead to several health problems including increased intracranial pressure and developmental impairments [19–22].

Organisms differ in the patency of cranial sutures. In zebrafish, all cranial sutures remain patent throughout the organism’s life [2]. Only the posterior interfrontal suture undergoes programmed fusion in the mouse [23]. In contrast in humans, with the exception of the metopic suture, which fuses during the first two postnatal years, cranial sutures normally do not fuse until the third or fourth decade of human life [24].

Several mechanisms involved in suture patency have been proposed, however, none of them have been studied in zebrafish. Using the mouse model, it has been proposed that the mechanism of suture patency relies on ossification fronts and sutural tissue being from different cell lineages [3]. Premature bone fusion was observed as a result of the incorrect mixing of cell populations at the border between the neural crest derived frontal bone and paraxial mesoderm-derived parietal bone in the *Twist1* mutant mouse [25]. Craniosynostosis was also induced by altering proliferation, apoptosis, and the rate of differentiation of the sutural mesenchyme, or in cells at the leading edges of ossifying bone [26–28].

The zebrafish model offers great potential for the study of suture patency and calvaria morphogenesis. However, this is an underdeveloped area of zebrafish research. Here, we aim to establish new protocols and to describe normal sutural tissue development and its maintenance in adult zebrafish. Based on our data and evaluation of the literature, we propose that zebrafish cranial vault development parallels the corresponding process in mammals.

## Materials and Methods

### Zebrafish Lines and Maintenance

All fish used in this study where raised and cared for in accordance with approved protocol by Ann & Robert H. Lurie Children's Hospital of Chicago Institutional Animal Care and Use Committee (IACUC# 13–008) and complied with NIH standards provided in the “Guide for the Care and Use of Laboratory Animals”. The AB line was used as wild type and *Tg(sp7*:*EGFP)*^b1212^ (ZDB-GENO-120413-2) transgenic line provided by Zebrafish International Resource Center.

### Fish harvesting

Fish were collected at various developmental stages, and the standard length (SL) was measured from the snout to the end of the hypuralia.

### Bone and cartilage staining

The procedure was performed as described before [29], however, staining solutions were applied sequentially for overnight incubation, beginning with Alcian blue and followed by Alizarin red. Crania were dissected, cleared in glycerol and mounted on a microscope slide. Photographs were taken using a Zeiss Stemi 2000 stereomicroscope equipped with a Zeiss Axiocam camera.

### Measuring cranial vault bones and suture development

**Fig 1:**
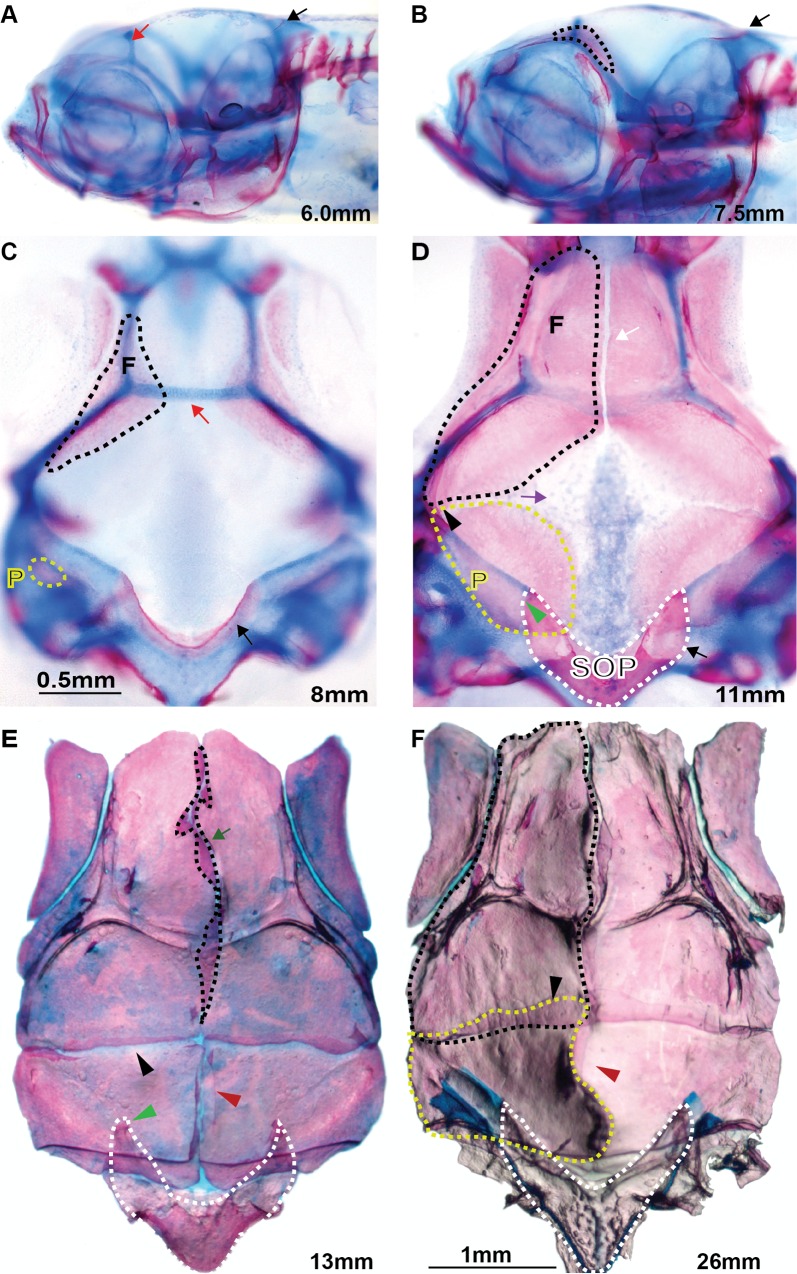
Developmental stages of zebrafish calvaria. (A) At 6 mm SL, ossification of the supraoccipital bone becomes visible as indicated by the black arrow; frontal bones are not detected yet. The cartilaginous epiphyseal bar is indicated by red arrow. (B) The frontal bones at 7.5 mm SL are outlined by the black dashed line. The supraoccipital bone is indicated by a black arrow in sections A-D. (C) At 8 mm SL, the frontal bones grow along the epiphyseal bar (red arrow) toward the midline, the parietal bones become visible as small radial patches symmetrically located on both sides of the cranium (outlined by the yellow dashed line). (D) At the 11 mm SL stage, the anterior part of interfrontal suture is developed (green arrow); frontal and parietal bones meet at the lateral side (black arrow head), initiating coronal suture formation, which progresses bilaterally toward the midline (purple arrow). The presumptive lambdoid suture develops between the parietal and supraoccipital bones (green arrowhead). (E) At 13 mm SL, all sutures are formed: the interfrontal (green arrow), coronal (black arrowhead), and sagittal suture (red arrowhead) and lambdoid (green arrowhead); the area specified by the black dotted line exemplify the measured area for morphometric study of the interfrontal suture. (F) Adult zebrafish cranium at age eight months, 26 mm SL. The black and yellow dashed lines indicate frontal and parietal bones respectively; the coronal and sagittal sutures are indicated by black and purple arrowheads respectively. (A, B) lateral view of the head; (C-F) Dorsal view of dissected calvaria stained with Alizarin red and Alcian blue. F–frontal bone, P–parietal bone, SOP–supraoccipital bone.

This study of cranial vault development was based on fish (n = 72) from a broad range of ages, varying from 5 mm larvae at 22 days post fertilization (dpf) to adults up to 35 mm at 526 dpf. Because siblings grown in identical environmental conditions demonstrate variable standard body lengths (SL), the SL value was used as a reliable indicator for developmental progress [30]. Fish were randomly selected, euthanized, and photographed for measurement. The following measurements were taken: cranial length, cranial width, and the projected areas of the cranial vault frontal, parietal, and supraoccipital bones. The projected areas of the interfrontal, coronal, sagittal and lambdoid sutures were also measured using the area of overlapping bones; an example is illustrated in Fig 1E by the black dotted line for area considered as the interfrontal suture and the red dotted line for the right coronal suture. A linear regression of measured areas on SL was calculated in STATA.

### Vital staining with Calcein green and Alizarin red to label mineralized matrix

Nine wild type zebrafish (21 dpf) with SL between 4.5 mm and 9 mm were used for live staining as described by [31]. 0.003% Alizarin red and 0.0025% Calcein green stains were applied sequentially for overnight incubations using water from the fish facility. Three different conditions were tested. In the first, Alizarin red stain was applied at 21 dpf (7.7 mm SL), followed by Calcein green 5 days later (8.1 mm SL), and Alizarin red was again added after 2 days (8.6 mm). In the second group, Alizarin red was applied at 21 dpf (8.0 mm SL) followed by Calcein green two weeks later (15.0 mm SL). In the third group, Alizarin red was applied at 42 dpf (16 mm SL) followed by Calcein 3 weeks later (26.0 mm SL). Finally, fish were rinsed from the staining solution and euthanized by overdose of Tricaine. Calvaria were dissected and mounted on a glass slide for imaging using the Zeiss V-8 Stereomicroscope, a Texas Red filter for detection of Alizarin red, and a GFP filter for Calcein green.

### Histological sections and light microscopy

Eleven wild type fish between 5 weeks (wpf) and 2 years of age (15 mm and 36 mm SL) were used for histological analysis. Paraffin sections were stained with Hematoxylin and Eosin (H&E) [32]. To visualize the interfrontal and sagittal sutures, a transverse plane of sectioning was used, while a sagittal section was chosen for coronal and lambdoid sutures. All fish were fixed in phosphate buffered 10% formalin overnight, followed by decalcification in 0.5M EDTA pH 8 for 1 to 4 days at room temperature, depending on fish size. Samples were processed using STP 120 Spin Tissue Processor—Thermo Scientific. The dehydration steps were conducted with 70%, 95%, 100% ethanol treatments, each repeated twice for 45 min. Xylene was applied twice for 45 min and paraffin (Thermo Scientific Histoplast Paraffin LP 8332) three times for 60 min. Specimens were embedded using the Microm EC350-2 tissue-embedding center. All tissue samples were sectioned at 5 μm using rotary microtome and stained with Hematoxylin and Eosin (H&E) [33] to evaluate morphological structures. Zeiss Axioplan2 Imaging Microscope equipped with an Axiocam HRc camera was used for imaging.

### Transmission Electron Microscopy (TEM)

Dissected calvaria from 14 wpf (n = 3) were fixed in 0.1M sodium cacodylate buffer pH 7.3 containing 2% paraformaldehyde and 2.5% glutaraldehyde for 24 hrs and decalcified in 0.5M EDTA for several days. The Cell Imaging Facility at Northwestern University Feinberg School of Medicine processed these samples for imaging.

### Picro sirius red staining for collagen

Cranial suture paraffin sections were stained using the Picro-sirius red (Direct red 80) technique as described by [34]. Haematoxylin was used to stain the nuclei and slides were mounted in DPX medium. In bright-field microscopy, collagen fibers appear red on a pale yellow background, and bright yellow or orange when DIC polarized light is used (Zeiss Axioplan2 microscope). Confocal laser scanning microscopy and Z-stack optical sections of the interfrontal suture were collected for three-dimensional reconstruction of collagen organization using Zeiss LSM510 and Zen-Zeiss software.

### Immunohistochemistry to detect SP7/GFP positive osteoblasts

Paraffin sections from *Tg(sp7*:*EGFP)*^*b1212*^ transgenic skulls at age 12 wpf (21 mm SL) were collected for immunohistochemistry. The primary anti-GFP antibody (Abcam) diluted to 1:500 and secondary Alexa Fluor 488 Donkey Anti-rabbit IgG diluted to 1:500 were used to detect the GFP reporter per manufacturer’s recommendations. DAPI counterstaining allowed for nuclei visualization. Specimens were observed using Zeiss 510 META Confocal Laser Scanning Microscope and 488 nm and 405 nm laser lines.

### RNAscope *in situ* hybridization

RNA probes, hybridization kits, and hybridization equipment were obtained from Advanced Cell Diagnostics. Formalin-fixed, paraffin-embedded μm sections were deparaffinized and treated per manufacturer’s recommendations, with small adjustments to the permeabilization and detection steps as follows. Slides were heated in Pretreat 2 reagent for 3 min at 101–104°C. Pretreat 3 solution was applied to slides and incubated at 40°C for 10 min in the ACD HybEZ Hybridization oven. Following amplification, the signal was detected for 20 min and counterstained with haematoxylin for nuclear visualization per manufacturer’s suggestions. The following probes were tested: *col1a1a*, *col2a1a*, *col10a1a*, *bglap*/*osteocalcin*, *fgfr1a*, *fgfr1b*, *fgfr2*, *fgfr3*, *twist2*, *twist3*, *runx2a*, *runx2b*, *sp7/osterix*, *spp1*, *foxq1a*, and *dapB* as a negative control. Each probe was tested in a minimum of three independent experiments. The weak expression within the sutural tissue was only considered positive when the expression in other tissue, e.g. head cartilages was obvious and as expected and the expression was noticeably higher than sections stained for the negative control.

We noticed that RNAscope *in situ*, provides a weaker signal for adult specimens than juvenile specimens. The longer specimens were treated with EDTA, which was used for bone decalcification, the weaker the measured signal.

## Results and Discussion

### Developmental schedule of cranial bones and sutures

As previously described, five major bones constitute the cranial vault: paired frontal bones, paired parietal bones, and a single supraoccipital bone. Our examination indicates that the earliest ossification of the zebrafish cranial vault is observed in the supraoccipital bone at 5.9 mm standard length (SL), as seen though Alizarin red staining. Ossification becomes noticeable as two thin lines form along the anterior edge of both arms of the “V shaped” cartilaginous framework (Fig 1A, 1B, 1C and 1D, black arrow) then progresses posteriorly as the two arms of the supraoccipital bone also extend laterally. The supraoccipital bone ossifies from a cartilage precursor. In contrast, frontal and parietal bones ossify intramembranously (n = 67).

**Fig 2:**
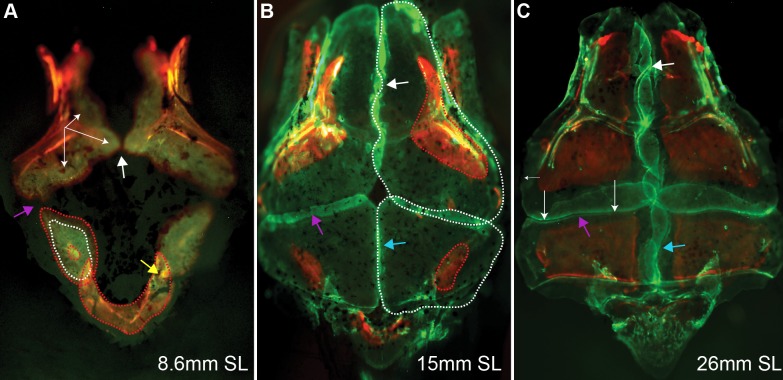
The growth pattern of calvaria bones as revealed by sequential staining with Alizarin red and Calcein green. (A) Initial directions of frontal bone growth (white arrows) and radial growth of the parietal bone are depicted after each vital staining by red and white dotted lines. The supraoccipital bone is depicted by the red dotted line. (B-C) The white, blue and purple arrows indicate developing sutures: interfrontal, sagittal and coronal, respectively. (B) The contour of frontal and parietal bones growth is outlined by red dotted lines labeling the first vital staining with Alizarin and by white-dotted lines the second treatment by Calcein green. (C) Posterior frontal bone advancement (long white arrows) revealed after second vital staining with Calcein green, and lateral growth of frontal bone (small arrow on the left side). Note the interdigitation of the frontal and parietal bones. Skulls were dissected and mounted for imaging from a dorsal view.

The ossification of the frontal bones is initiated symmetrically at the lateral edge of the posterior *taenia marginalis* (Fig 1A). Then it progresses anteriorly along the *taenia marginalis* and medially along the underlying epiphyseal bar (Fig 1A and 1C red arrow), which, according to the recent transgenic studies [5], demarcates the posterior end of the NC contribution to the frontal bone. The opposite edges of the growing frontal bones come first into proximity at the level of the epiphyseal bar at around 10 mm SL (Fig 2A, blue arrow). Then, the juxtaposition of frontals progresses anteriorly along the midline, reaching the end-to-end position at around 11 mm SL (Fig 1D, white arrow). The posterior frontal bones of the NC origin start to approach each other along the midline, starting from the epiphyseal bar. Frontal bones reach full association at about 12.5 mm SL.

Similar results were obtained when sequential live staining of bones with Alizarin red and Calcein green was applied, however, it was also obvious that frontal bones also expand laterally as the skull develops, though at a much slower rate (Fig 2A–2C). In addition this experiment revealed the areas of bone thickening, visualized as yellow, when both stains were absorbed.

**Fig 3:**
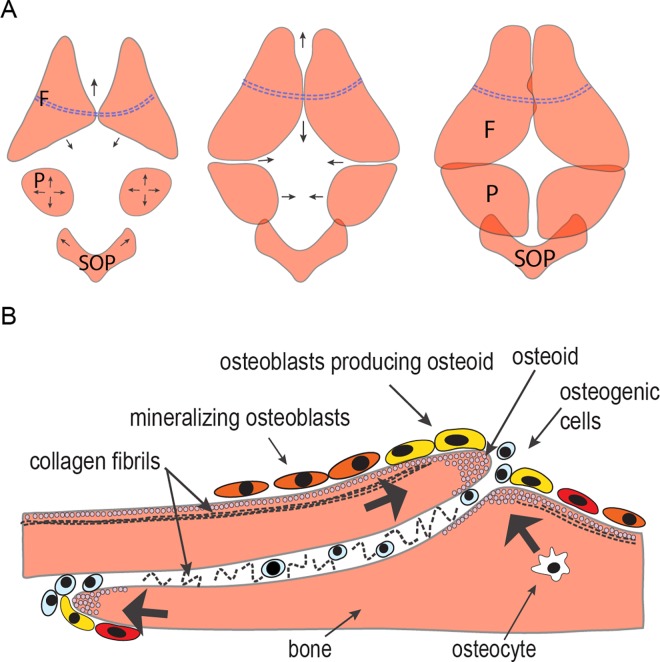
Schematic representation of the cranium development. (A) Developmental steps of frontal (F), parietal (P) and supraoccipital (SOP) bones growth; directions of bones growth are indicated by arrows; dotted line indicates the epiphyseal bar. (B) Cellular and matrix components making up the interfrontal suture. Black arrows indicate the direction of bone growth and mineralization.

As the skull continues to grow and the frontal and parietal bones encounter each other on the lateral side of the skull, an elastic junction is formed between them, known as the coronal suture (Fig 1D, the green arrow indicates the developing interfrontal suture; the black arrowhead shows the coronal suture and schematic representation Fig 3A).

The ossification of the parietal bone is initiated at the lateral center and was detected at 7.2 mm SL. Two parietal bones develop symmetrically on either side of the skull to form the posterior roof of the cranium (Fig 1C and 1D parietal bone indicated by yellow dashed line). Sequential live staining with Alizarin red and Calcein green revealed two small patches of intramembranous ossification that grow radially with new bone tissue being added at their edges, analogous to the process described in rodents and humans (Fig 2A, parietal bone primodium labeled with red and white dashed lines). This initially radial ossification, observed as circumferentially deposited layers of red and green stains incorporated into newly deposited bone matrix (indicated by doted lines) is followed by asymmetrical ossification, which progresses anteriorly toward the presumptive coronal suture (Fig 2A, red arrow) and later medially towards the presumptive sagittal suture (Fig 1B–1D). Development of the first cranial suture, the lambdoid, is initiated when the growing anlage of the parietal bone becomes associated with the supraoccipital bone (Fig 1D, green arrowhead). In zebrafish, as in mammals, the lambdoid suture is uniquely formed between the supraoccipital bone of endochondral origin and the intramembranously formed parietal bone. Live sequential bone staining with Alizarin red and Calcein green suggests that, in zebrafish, the lambdoid is the first suture initiated in the developing cranial roof. A similar suture was identified in *Xenopus* [35].

At 8 mm SL, the parietal and frontal bones juxtapose at the lateral edge of the skull, initiating the formation of the coronal suture (Fig 1D, black arrowhead). The frontal bone progressively overlaps the anterior edge of the parietal bone, advancing symmetrically towards the midline. Formation of the sagittal suture between two parietal bones is initiated by 13 mm SL (Fig 1E and 1F, red arrowheads and Fig 2B and 2C, blue arrows). Similarly, in rodents, frontal and parietal bones grow from an initially basolateral position upwards toward the apex of the skull [4]. Our results suggest that in zebrafish, as in humans, each parietal bone develops from a single ossification center. After the initial phase of radial advancement, the parietal bones also undergo directional growth towards the presumptive coronal and sagittal sutures. Zebrafish calvarial roof development significantly differs from that of *Xenopus*. The frontal and parietal bones are fused in frogs. They form one frontoparietal bone, which also initiates laterally, but develops in a posterior-to-anterior direction [35].

To summarize, development of the calvarial roof in zebrafish follows an intricate pattern, which leads to the formation of a structure that closely resembles the mammalian cranium, although the overall shape of the skull is dramatically different. Schematic representation of cranial roof development in zebrafish is presented in Fig 3A.

### Cranial vault bone and suture growth show linear relationship with standard length

**Fig 4:**
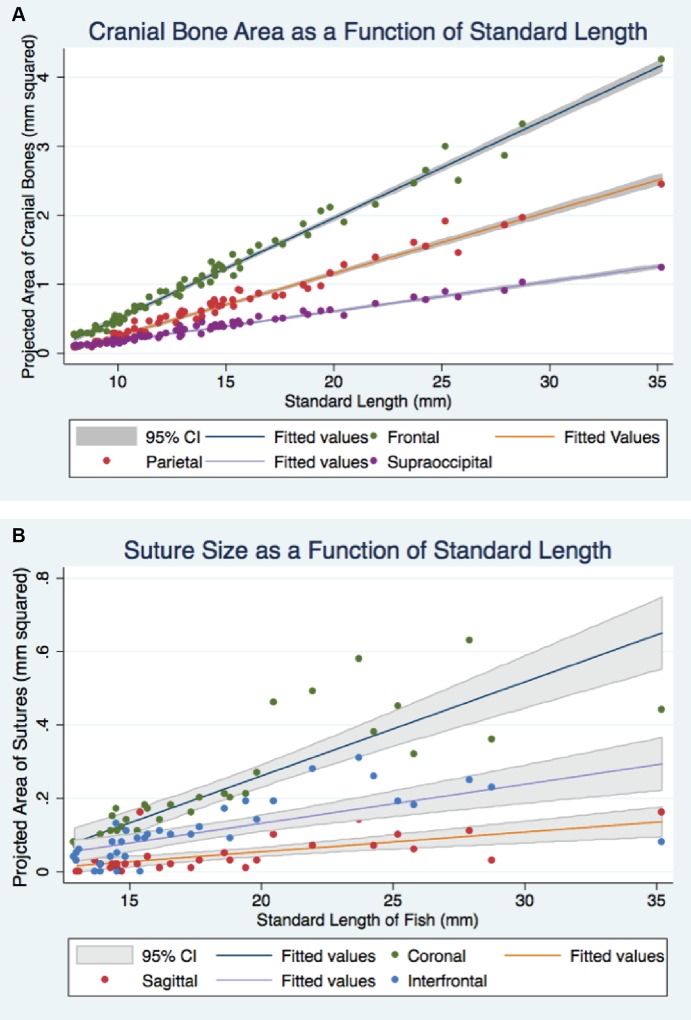
Correlation of growing cranial bones and sutures with increasing standard length. (A) Each cranial bone demonstrates a unique, strong linear correlation with standard length. The frontal bone shows the most rapid growth rate, followed by the parietal and the supraoccipital bone. (B) The coronal suture shows the strongest correlation with standard length.

To assess skull growth and the potential relationship between standard length, representing the developmental stage of fish, and projected areas, representing size of the cranial bones and sutures, we performed a linear regression. As described in the Methods, measurements were collected from n = 72 fish of different age, as represented by SL. This is shown graphically by scatter diagram in Fig 4 and Table 1.

**Table 1:** Regression table for a scatterplot and regression graphs presented for cranial bone and suture.

	Frontal Average [mm^2^]	Parietal Average [mm^2^]	SOP [mm^2^]	Coronal [mm^2^]	IF [mm^2^]	Sagittal [mm^2^]
Standard Length (mm)	0.146*** (0.00202)	0.0902*** (0.00166)	0.0432*** (0.00072)	0.0258*** (0.0025)	0.0112*** (0.0019)	0.00537*** (0.00114)
Constant	-0.952*** (0.0306)	-0.649*** (0.0252)	-0.255*** (0.0111)	-0.256*** (0.0473)	-0.0938* (0.0354)	-0.0531* (0.0217)
*N*	72	72	72	38	38	34
*R*^2^	0.987	0.977	0.980	0.740	0.489	0.411

Standard errors in parentheses

* *p* < 0.05

** *p* < 0.01

*** *p* < 0.001

CI- confidence interval, SOP–supraoccipital bone, IF- interfrontal suture

We observed that as the zebrafish continues to grow through its lifetime (we estimate an average of 0.34 mm per week), the growth of cranial bones and sutures, as measured by the projected area, follows a linear relationship. Increases in the width of the coronal suture, which develops in parallel to body length, are most strongly correlated with an increase in SL. By contrast, the sagittal suture, which develops perpendicular to the body length axis, exhibits the weakest, although still positive, correlation. These values can be used to estimate pathological changes in the size of cranial bones and sutures in future models of craniosynostosis in zebrafish. Our analysis also confirms visual observations that the frontal bones are the most rapidly developing bones, followed by the parietal and supraoccipital bones.

### Cranial suture appearance

**Fig 5:**
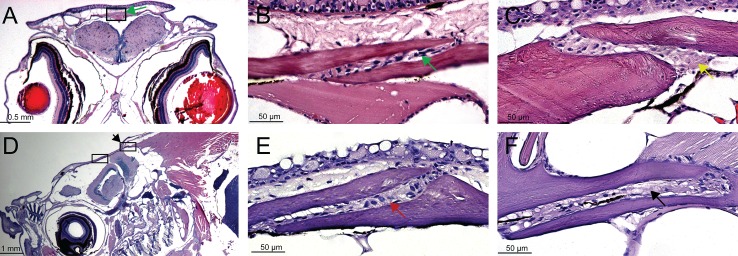
Histological examination of cranial sutures in adult zebrafish. H&E stained transverse sections (A-C) and sagittal sections (D-E) of skulls. (A) The interfrontal suture (boxed) and presented in (B) higher magnification; green arrows indicate the interfrontal suture between overlapping frontal bones. (C) The sagittal suture (yellow arrow) observed between two parietal bones. (D) The sagittal plane of sectioning revealed the coronal (left box) and lambdoid sutures (right box, black arrow). (E) The posterior frontal bone overlapping the anterior portion of the parietal bone, with the coronal suture formed between them (red arrow). (F) The lambdoid suture (black arrow) separates the parietal (upper plate) and the supraoccipital bone (lower plate).

Cranial sutures are deformable joints formed between bones bridged by collagen fibers. Suture morphology may range from straight-edged to interdigitated. The elaborate pattern of interdigitation is especially apparent along the midline and is prevalent in the interfrontal suture. However, the sutural outline on the surface of the skull, as visualized by whole mount Alizarin red staining, may not reflect suture morphology as accurately as do perpendicular sections and histological staining (Fig 5A–5F). Indeed our observations suggest that only perpendicular sections of studied suture can expose patency or bone fusion.

To examine the anatomy of zebrafish cranial sutures, we applied H&E histological staining to sections of skulls embedded in paraffin. Transverse and sagittal sections from animals at different ages (5 wpf juveniles to 2 year old adult zebrafish) were evaluated. Nuclei positive for hematoxylin were observed within the sutures and in cells lining the cranial bone plates (Fig 5). The interfrontal, coronal, sagittal and lambdoid sutures (Fig 5B) were examined and all provide strong evidence for maintained suture patency in zebrafish. Examination of the interfrontal suture revealed that the osteogenic front of the frontal bones can split during development and the opposite bone grows in-between. No directional preference for the side where the split occurs was observed. The sagittal suture exhibited an overlapping pattern, either as two parietal bones sliding one over the other without a specific direction (example Fig 2C, blue arrow), or two bones positioned end-to-end (Fig 5C). The coronal suture forms in a uniform pattern where the frontal bone consistently overlaps dorsally over the anterior part of the parietal bone (Fig 2B and 2C, purple arrow and Fig 5E, red arrow). We predict that the continuous growth of the zebrafish body requires a mechanism that provides concomitant and proportional increase in skull growth. Continual growth and lifelong patency of the cranial suture could provide such a mechanism. The reason for the elaborate pattern of the interfrontal suture is difficult to explain. However computational analysis conducted by Jasinoski et al. [36] suggests that interdigitated sutures absorb more energy than straight ones, hence providing better protection of the brain.

### Cellular genetic markers identified in the suture

**Fig 6:**
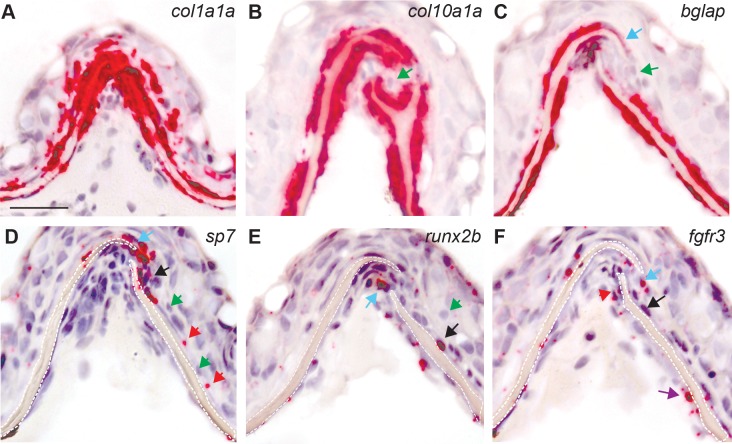
RNA detection for genetic markers of osteoblasts in juvenile animals assessed using RNAscope *in situ* hybridization on paraffin section. (A-F) Sequential sections (4 μm) of the interfrontal suture collected from juvenile zebrafish at age of 6 wpf. The expression of individual genes is visualized in red, counterstained with haematoxylin for nuclei in purple; black arrows examples of positive expression, all green arrows indicate negative for expression cells. (A) The expression of *col1a1a* is present in all cells. (B) The green arrow indicates cells that are negative for *col10a1a* expression in the mid-suture domain. (C) Expression of *bglap* weaker at the tips of frontal bones as indicated by blue arrow. (D) Expression of *sp7* at osteogenic fronts (blue arrow) and along the frontal bone (red arrow), (E) *runx2b* at the osteogenic front (blue arrow) and along the bone (black arrow). (F) *fgfr3* expression observed in osteogenic front (blue arrow) and along the frontal bones (black and purple arrows). The scale bar represents 20 μm.

**Fig 7:**
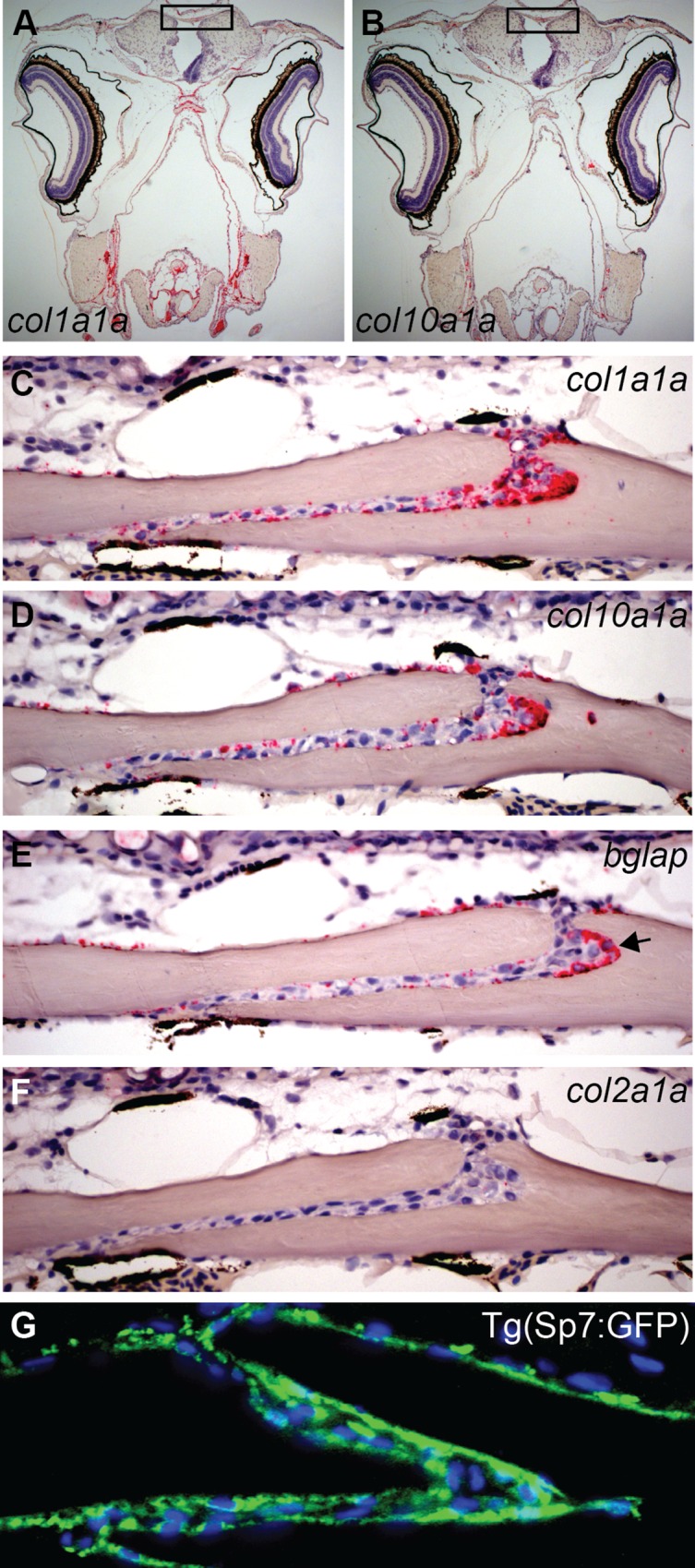
RNAscope in situ hybridization and IHC for GFP performed for adults at 12 wpf. (A-F) Sequential sections (4 μm) of interfrontal suture expression. Names of genes tested by RNAscope in situ hybridization are labeled on each image. (A, B) Representative images of the skull taken at low magnification (5x) that visualize entire expression pattern of (A) *col1a1a* and (B) *col10a1a*. (C-F) Images of interfrontal sutures and the expression of representative genes as labeled on each image. (G) Immunohistochemistry for sp7 observed in the interfrontal suture using a GFP reporter in the Tg(sp7:EGFP)^b1212^ transgenic line. Transverse paraffin section, GFP positive cells (green) and nuclei stained with DAPI (blue).

The mechanisms underlying the morphogenesis of the zebrafish cranial roof are not well known. The literature on rodents describes a population of cells between the osteogenic fronts of developing bones and corresponding sutures with different origins and functions. These are mesenchymal cells, pre-osteoblasts, and gradually maturating osteoblasts involved in bone formation, as well as osteoclasts involved in bone remodeling [37]. Initially, using RT-PCR on total RNA isolated from calvaria of zebrafish age 4–6 weeks, we tested for expression of different genes associated with suture development in mammals. The RT-PCR was positive for all tested genes: *fgfr1a*, *fgfr1b*, *twist 1a*, *twist 1b*, *twist2*, *twist3*, *msx2*, *wnt5a*, *wnt5b*, *foxd1* (data not shown) so we developed RNA *in situ* hybridization on paraffin sections to assess the expression patterns within the sutural tissue (Figs 6 and 7).

The following genes were tested: *col1a1a*, *col2a1a*, *col10a1a*, *bglap*, *twist2*, *twist3*, *sp7*, *runx1a*, *runx1b*, *spp1*, *fgfr1a*, *fgfr1b*, *fgfr2*, *fgfr3*, *foxq1a* on transverse sections of the skull taken from juvenile fish (6 wpf) and adults (14 wpf). RNAscope *in situ* hybridization for *col1a1a* encoding Type I collagen alpha 1a indicates very abundant expression within the sutural tissue of juvenile and adults. With age however, the number of cells lining the osteogenic fronts declines, and so does the expression of *col1a1a* in these cells and in the suture (Figs 6A, 7A and 7C). In mammals, Type I collagen represents approximately 95% of the entire collagen content of bone and about 80% of the total bone protein [38] and synthesis of Type I collagen precedes the expression of *Runx2*, considered to be the earliest determinant of osteoblast differentiation in mice [39].

The expression of *col2a1a*, which encodes for fibrillar type II alpha1 collagen, was barely detected within the interfrontal suture, in juvenile and adults zebrafish. However, as a major component of the cartilage, the expression of *col2a1a* was detected in head cartilages at a dramatically higher level and on the same sections supporting our conclusion that the expression of *col2a1a* in the interfrontal stature is either negative or on the border of the sensitivity of the RNAscope method (Fig 7F and S1–S3 Figs).

In juvenile animals, the expression of *col10a1a* encoding Collagen type X alpha 1a has a similar pattern to *col1a1a*, with the exception of a small gap in the expression observed in the middle of the suture in cells that separate the leading edges of frontal bones (Fig 6B, green arrow). Based on their position, we presume that these *col10a1a* negative cells represent mesenchymal cells or pre-osteoblasts. In higher vertebrates, *Col10a1* is considered an exclusive marker for hypertrophic chondrocytes [40]. Indeed, zebrafish hypertrophic chondrocytes were positive for *col10a1a* RNA on all tested slides (n = 15). We found that cells located at the immediate periphery of cartilaginous structures, like the perichondrium of the quadrate, were positive for *col10a1a* (S2 Fig). This observation is consistent with previously described [41] expression of *col10a1* in mature chondrocytes and in perichondral cells of ceratohyals as well in osteoblasts of dentaries [41]. The expression of *col10a1a* was also described in the forming parasphenoid, ectopterygoid, and operculum formed by intramembranous mechanism, devoid of cartilage precursors as the earliest in the craniofacial region during intramembranous bone growth and development [42]. Our *in situ* hybridization on adjacent slides suggests that during the initial steps of suture development, when the frontal bones come into proximity, there is a group of cells located in the central part of the sutural tissue that are negative for collagen *col10a1a* (Fig 6B, green arrow). These cells are positive for *col1a1* and likely to be positive for *sp7* and *runx2b*, (Fig 6A, 6D, 6E and 6F). Based on this observation we speculate that, unlike in higher vertebrates, hypertrophic chondrocytes and cranial osteoblasts at early stages of maturation as well as mature osteoblasts express *col10a1a*. In the study of vertebral column development in medaka based on analysis of the GFP reporter in transgenic *col10a1*:*nlGFP*, it was proposed that *col10a1a* expression in the osteoblasts precedes the ossification of the vertebral column [43].

When *bglap* expression was analyzed, we found that the mesenchymal cells are negative for the expression and only a few cells in the osteogenic fronts express *bglap* (Fig 6C, blue arrow). The expression is seen in osteoblasts lining the inner and outer surfaces of the frontal bones but located further from the osteogenic fronts. In adults (Fig 7E), the expression is excluded from the inner surface of frontal bones and appears weaker on the outer surface; transcripts are easily detected within the sutural tissue, especially in the bone cavity (black arrow) where we presume the bone is also formed. The presence of *bglap* positive cells lining the surfaces of both frontal bones correlates with an intense process of bone thickening in juvenile animals. In young adults (14 wpf) *bglap* expression diminishes from the outer surface of the frontal bones and almost disappears from the inner surface. The process of ossification slows down around this age as well. *Bglap* expression is recognized as a marker of mature osteoblasts [44, 45] and the expression pattern seen in zebrafish agrees with accumulation of mRNA in areas of the bone formation.

The examination of the expression of *sp7*, an osteoblast specific transcription factor, revealed an almost reverse pattern of that which was observed for *bglap*. The expression of *sp7* was mainly detected in the osteogenic fronts, and the suture mesenchyme in cells which were negative for *bglap* (Fig 6C and 6D). However, a small overlapping area with *bglap* expression was seen in the osteogenic fronts (blue arrows) and in a few cells lining the outer side of the frontal bone where *bglap* positive cells reside (Fig 6C, red arrows). In adults, the *sp7* transcripts were not reliably detected in tested sections (data not shown). Accordingly, we examined the population of cells positive for *sp7*, by immunohistochemistry against the GFP reporter on paraffin sections obtained from *Tg(sp7*:*EGFP)*^*b1212*^ transgenic skulls (Fig 7G). In this line, the expression of the GFP reporter occurs concurrently with Sp7 [46, 47]. This result revealed that in this transgenic line, GFP positive cells dominated the sutural tissue (Fig 7G) covering both sides of frontal bones. We assume that the stability of the GFP reporter could partially explain this result. Studies of murine osteoblasts suggest that expression of *Sp7* is initiated as soon as the mesenchymal cells enter the osteoblast lineage and becomes stronger with osteoblast differentiation [48, 49]. Our data from juvenile sections of the interfrontal suture suggest that in zebrafish, *sp7* is strongly expressed by mesenchymal and osteogenic front cells and suggests that the transcription decreases with osteoblast maturation. The contradictory results between *in situ* data and immunohistochemistry are difficult to explain; perhaps more systematical, comparative analysis of *sp7* expression in *Tg(sp7*:*EGFP)*^*b1212*^ transgenic line is needed.

Runx2 is essential for the commitment of mesenchymal cells into the osteoblastic lineage and is dynamically expressed during osteoblast maturation. RUNX2 activates the expression of some bone matrix protein genes, and keeps the osteoblasts in an immature stage. *Runx2* expression must be downregulated for differentiation into mature osteoblasts [40, 50, 51]. Increased *Runx2* expression coincides with premature suture closure in many models of craniosynostosis [52].

Zebrafish has two homologues of *Runx2*, *runx2a* and *runx2b*, and both genes were tested in juveniles and adults. The *runx2b* expression seams stronger than *runx2a* and was detected in mesenchymal cells and tips of the osteogenic fronts (Fig 6E, S1 Fig) transcripts extend into cells lining both sides of the bones (Fig 6E, black arrow); both *runx2a* and *runx2b* were detected in the head cartilage elements, providing a good internal control (S2 Fig). In the mouse *Runx2* expression partially overlaps with *Fgfr2* and *Fgfr1* [4, 28, 53] therefore we tested for these genes expression on sequential sections. The *fgfr3* [37, 54] has the highest accumulation of transcripts, detected in the osteogenic fronts as well in cells lining the frontal bone, including presumed mature osteoblasts (Fig 6F, purple arrow). Transcripts for *fgfr1a*, *fgfr1b*, *fgfr2* were difficult to distinguish from controls, these results were moved to supplementary data (S1 and S2 Figs). In adults, a majority of the *runx2b* expression is restricted to osteoblasts within the sutural tissue. A very weak signal was detected on the outer surface of the frontal bones (data not shown).

The following genes were also tested: *spp1*, *twist2*, *twist3*, *foxq1a* though the expression levels within the sutural tissue were questionable (S1 Fig). For *spp1* we found a few potentially positive cells in the mid-suture area and in cells lining the inner side of the bone (S1 Fig, black arrows). The *spp1* is a extracellular matrix protein involved in the bone remodeling process [55] and is known to be a mineralization inhibitor that binds to the apatitic mineral crystals in bones [56] *in vivo* and in osteoblast cultures [56–58]. The *foxq1a* [59] hypothetical expression was detected in the middle of sutural tissue, presumably in mesenchymal cells that might also express *runx2b* and *col1a1a* (S1 Fig, black arrow) but not *col10a1a*, suggesting that this transcription factor might be expressed by less differentiated osteoblasts or presumably their precursors.

Signals of *twist2* and *twist3* [14] were below RNAscope detection level (S1 Fig). In murine model the expression of *Twist* is observed in the midsutural mesenchymal cells and overlaps with *Fgfr2*, representing the most immature (proliferating) osteogenic cells [14, 53, 60]. *Twist* +/- mice exhibit faster mineralization and abnormalities in cranial vault development [61]. As expression of zebrafish homologues is hypothetical it is difficult to conclude from our data whether proliferating osteoprogenitors express twist genes.

### TEM microscopy of the interfrontal suture

**Fig 8:**
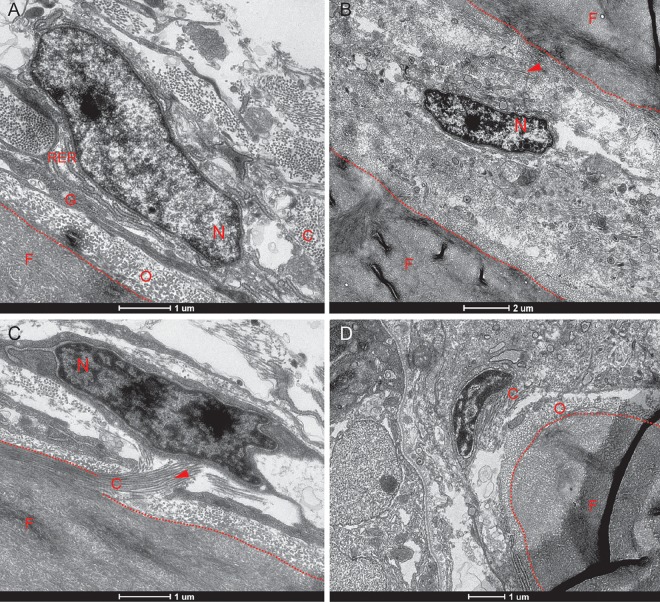
Transmission electron micrographs show various osteogenic cells within the interfrontal sutures. The calvarium was isolated from adult zebrafish at 14 wpf. (A) An osteoblast in direct contact with the osteoid of the upper frontal plate, indicative of ongoing ossification. (B) A mid-suture osteoblast not attached to bone. (C) The longitudinal organization of collagen fibrils along the frontal bone. (D) The tip of the lower plate of the frontal bone, with adjacent associated osteoblast. Abbreviations: C—collagen fibrils, F—frontal bone, G—Golgi apparatus, N—nucleus, O–osteoid, RER–rough endoplasmic reticulum; arrows indicate collagen fibrils.

Our histological and gene expression studies indicate that the cranial suture has a complex architecture, which includes osteogenic cells at different steps of maturation surrounded by an elaborate network of extracellular matrix (ECM). The literature also suggests that the morphology of osteogenic cells correlates with their function [62, 63]. Therefore, to assess the ultra-structural level of interfrontal suture organization, we applied TEM microscopy. Transverse sections of the interfrontal suture were collected from 14 wpf adults (Fig 8). TEM micrographs confirmed the presence of osteoblasts lining the outer surface of frontal bones. These cells have a prominent nucleus and an elaborate rough endoplasmic reticulum (RER) and Golgi apparatus, indicative of intensive protein synthesis (Fig 8A). We predict that these are osteoblasts, producing ECM components such as collagen type I and collagen type X, as both transcripts were identified by in situ hybridization as abundantly present. In the mid-suture area, cells were found at a distance from the bone, surrounded by fibrils of collagen and other components of ECM (Fig 8B). Interestingly, in some osteoblasts attached to the bone, we noticed electron dense granules inside of mitochondria (data not shown). It has been described that inorganic components like calcium phosphate could accumulate in mitochondrial granules of mineralizing osteoblasts [64]. We also noticed the presence of crystallites in the osteoid being in direct contact with such osteoblasts, suggesting that these are probably mineralizing osteoblasts. The TEM micrographs provided evidence of abundant Type I collagen, organized in characteristically banded, periodic-structured fibrils, which occasionally form bundles. Longitudinally organized collagen fibrils were observed along the frontal bone plates (Fig 8C). Characteristically banded collagen fibrils were also observed in the unmineralized layer of the ECM (Fig 8A and 8D).

### Collagen organization within the suture

**Fig 9:**
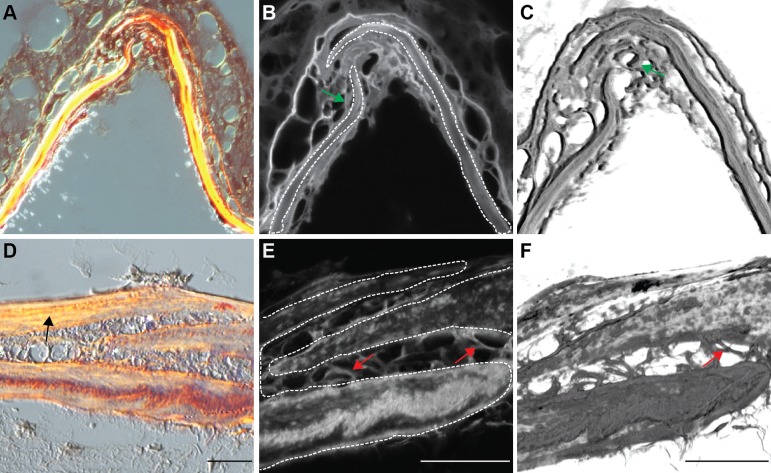
Picro sirius red staining of transverse sections of the skulls. Plane polarized light microscopy for (A, B, C) juvenile fish (13.4 mm SL) and (D, E, F) young adult fish at age of 12 wpf respectively, at 40x magnification. (B, E) confocal microscopy of the same sections at 100x magnification, and (C, F) 3D rendered confocal images at 100x magnification. The longitudinal organization of collagen fibrils is indicated by a black arrow (D). The frontal bones are outlined by white dashed lines (B, E). The left frontal plate developed a split end and the right frontal bone grew in between. The red arrow indicates fibers connecting two frontal bones; the green arrow indicates fibers that grown in between the cells. Scale bars are 20 μm.

Collagen proteins, a principal component of the extracellular matrix, play a dominant role in the preservation of connective tissue structures, providing strength and flexibility. To assess the organization of collagen fibrils in the zebrafish interfrontal suture, we used Picro sirius red staining on transverse paraffin sections. This method relies on an optical property of collagen called birefringence [65]. In polarized light, the larger collagen fibers, mostly Type I, are bright yellow or orange, and the thinner ones, including reticular fibers, are green [66, 67]. We have tested juvenile zebrafish at age 6 wpf and adult zebrafish at age 14 wpf (Fig 9). No apparent orthogonal lattice or lamellar organization of collagen fibers was observed within the suture (n = 18). When analysis was extended to confocal microscopy and three-dimensional reconstruction of the optical sections, similar results were obtained (Fig 9C and 9F). Frequently, we observed randomly organized collagen fibers connecting two frontal bones (Fig 9E and 9F, red arrows) or fibers that intervene among cells that reside in the interfrontal suture (Fig 9B and 9E, green arrows). Both types of analysis suggest that as zebrafish age, the distance between collagen fibers increases. This could result in a gradual deficit of suture flexibility provided by collagen. It was proposed that collagen fibrils become organized into an orthogonal lattice within the normally fusing frontal suture in rats and pathologically fusing suture in humans. In contrast, patent sagittal suture in rats and healthy sagittal suture in humans maintain a random organization of collagen fibers [68]. We concluded that in zebrafish, as in higher vertebrates, random organization of collagen fibers is typical within the interfrontal suture. We observed the apparent longitudinal organization of collagen fibrils along the frontal bone plates (Fig 9D, black arrow), consistent with organization found in higher vertebrates. The Picro sirius red staining approach could be helpful in assessing collagen organization in the fusing suture of a mutant model of craniosynostosis.

## Conclusions

In this study, we have analyzed calvarial bone and suture growth in zebrafish, starting from the stage when calvarial development is initiated through adulthood. Our results support past findings that, in general, the architecture of zebrafish calvaria is similar to that of mammals, with the key difference being that zebrafish sutures maintain lifelong patency. Using different methods of analysis, we provide new insight into the cellular composition of cranial sutures in zebrafish. The TEM data and genetic marker analysis provide evidence that osteoblasts that are located at the leading edge of the osteogenic front are presumably involved in the synthesis of major protein components of ECM, such as collagens. Following them are mature osteoblasts positive for osteocalcin, presumably involved in the mineralization of the osteoid. In the mid-suture area, we identified cells that are detached from the bone and lack elaborate RER or Golgi apparatus. These are cells that primarily express *col1a1a*, *sp7*, *runx2b*, *fgfr3*, hypothetically also *twist3* and *foxq1a*, which is a novel gene for suture study. We assume they are less differentiated osteoblasts and presumably some of them represent osteoblast precursors. A schematic representation of our data is presented in Fig 3. Our findings provide a foundation for the use of zebrafish as a model of calvarial bone and suture dysfunction.

## Supporting Information

S1 Fig RNA detection for genetic markers of osteoblasts in juvenile animals assessed using RNAscope *in situ* hybridization on paraffin section. (A-I) Sequential sections (4 μm) of the interfrontal suture collected from juvenile zebrafish at age of 6 wpf. The expression of individual genes is visualized in red, counterstained with haematoxylin for nuclei in purple; black arrows indicate assumed positive expression. The expression of following genes is shown: (A) *col2a1a*, (B) *runx2a*, (C) *spp1*, (D) *fgfr1a*, (E) *fgfr1b*, (F) *fgfr2*, (G) *twist2*, (H) *twist3*, (I) *foxq1a*. The scale bar represents 20 μm.
(TIF)

S2 Fig The RNA *in situ* detection in ceratohyal cartilage at 6 wpf evaluated by RNAscope technique. The images of the ceratohyal cartilage (dotted box and black arrowhead in A) collected from the tissue sections used for the interfrontal suture analysis shown in Fig 6 and S1 Fig. The expression of individual genes is visualized in red, counterstained with haematoxylin for nuclei in purple. The expression pattern of (A, B) *col1a1a*, (C) *bglap*, (D) *fgfr1a*, (E) *fgfr1b*, (F) *fgfr3*, (G) *runx2a*, (H) *runx2b*, (I) *spp1*.
(TIF)

S3 Fig The RNA detection in the palatoquadrate cartilage at 6wpf. Both, the left and right palatoquadrate cartilages are shown as follows: *fgfr3*, *bglap*, *cyp26a*, *col1a1a*, *col2a1a*, *col101a*. The same tissue sections were analyzed for gene expression in the interfrontal suture as presented in Fig 6 and S1 and S2 Figs.
(TIF)

## References

[1] Cubbage CC, Mabee PM. Development of the cranium and paired fins in the zebrafish Danio rerio (Ostariophysi cyprindae). J Morphol 229 1996;229:121–60. 29852585 10.1002/(SICI)1097-4687(199608)229:2<121::AID-JMOR1>3.0.CO;2-4

[2] Quarto N, Longaker MT. The zebrafish (Danio rerio): a model system for cranial suture patterning. Cells, tissues, organs. 2005;181(2):109–18. Epub 2006/03/15. 10.1159/000091100 . 16534205

[3] Morriss-Kay GM, Wilkie AO. Growth of the normal skull vault and its alteration in craniosynostosis: insights from human genetics and experimental studies. Journal of anatomy. 2005;207(5):637–53. Epub 2005/11/30. 10.1111/j.1469-7580.2005.00475.x 16313397 PMC1571561

[4] Yoshida T, Vivatbutsiri P, Morriss-Kay G, Saga Y, Iseki S. Cell lineage in mammalian craniofacial mesenchyme. Mechanisms of development. 2008;125(9–10):797–808. Epub 2008/07/12. 10.1016/j.mod.2008.06.007 . 18617001

[5] Kague E, Gallagher M, Burke S, Parsons M, Franz-Odendaal T, Fisher S. Skeletogenic fate of zebrafish cranial and trunk neural crest. PloS one. 2012;7(11):e47394 Epub 2012/11/17. 10.1371/journal.pone.0047394 23155370 PMC3498280

[6] Szabo-Rogers HL, Smithers LE, Yakob W, Liu KJ. New directions in craniofacial morphogenesis. Developmental biology. 2010;341(1):84–94. Epub 2009/11/28. 10.1016/j.ydbio.2009.11.021 . 19941846

[7] Yelick PC, Schilling TF. Molecular dissection of craniofacial development using zebrafish. Critical reviews in oral biology and medicine. 2002;13(4):308–22. Epub 2002/08/23. . 12191958 10.1177/154411130201300402

[8] Jiang X, Iseki S, Maxson RE, Sucov HM, Morriss-Kay GM. Tissue origins and interactions in the mammalian skull vault. Developmental biology. 2002;241(1):106–16. Epub 2002/01/11. 10.1006/dbio.2001.0487 . 11784098

[9] Opperman LA. Cranial sutures as intramembranous bone growth sites. Developmental dynamics: an official publication of the American Association of Anatomists. 2000;219(4):472–85. Epub 2000/11/21. 10.1002/1097-0177(2000)9999:9999<::AID-DVDY1073>3.0.CO;2-F . 11084647

[10] Feng W, Choi I, Clouthier DE, Niswander L, Williams T. The Ptch1(DL) mouse: a new model to study lambdoid craniosynostosis and basal cell nevus syndrome-associated skeletal defects. Genesis. 2013;51(10):677–89. Epub 2013/07/31. 10.1002/dvg.22416 23897749 PMC3918964

[11] Hatch NE. FGF signaling in craniofacial biological control and pathological craniofacial development. Critical reviews in eukaryotic gene expression. 2010;20(4):295–311. Epub 2010/01/01. . 21395503 10.1615/critreveukargeneexpr.v20.i4.20

[12] Khonsari RH, Delezoide AL, Kang W, Hebert JM, Bessieres B, Bodiguel V, et al Central nervous system malformations and deformations in FGFR2-related craniosynostosis. American journal of medical genetics Part A. 2012;158A(11):2797–806. Epub 2012/09/19. 10.1002/ajmg.a.35598 . 22987770

[13] Ornitz DM. FGF signaling in the developing endochondral skeleton. Cytokine & growth factor reviews. 2005;16(2):205–13. Epub 2005/05/03. 10.1016/j.cytogfr.2005.02.003 15863035 PMC3083241

[14] Rice DP, Aberg T, Chan Y, Tang Z, Kettunen PJ, Pakarinen L, et al Integration of FGF and TWIST in calvarial bone and suture development. Development. 2000;127(9):1845–55. Epub 2000/04/06. . 10751173 10.1242/dev.127.9.1845

[15] Ishii M, Sun J, Ting MC, Maxson RE. The Development of the Calvarial Bones and Sutures and the Pathophysiology of Craniosynostosis. Curr Top Dev Biol. 2015;115:131–56. 10.1016/bs.ctdb.2015.07.004 . 26589924

[16] Levi B, Wan DC, Wong VW, Nelson E, Hyun J, Longaker MT. Cranial suture biology: from pathways to patient care. The Journal of craniofacial surgery. 2012;23(1):13–9. Epub 2012/02/18. 10.1097/SCS.0b013e318240c6c0 . 22337368

[17] Wilkie AO. Craniosynostosis: genes and mechanisms. Human molecular genetics. 1997;6(10):1647–56. Epub 1997/01/01. . 9300656 10.1093/hmg/6.10.1647

[18] Kolar JC. An epidemiological study of nonsyndromal craniosynostoses. The Journal of craniofacial surgery. 2011;22(1):47–9. Epub 2010/12/29. 10.1097/SCS.0b013e3181f6c2fb . 21187784

[19] Kabbani H, Raghuveer TS. Craniosynostosis. American family physician. 2004;69(12):2863–70. Epub 2004/06/30. . 15222651

[20] Cohen MM Jr. No man's craniosynostosis: the arcana of sutural knowledge. The Journal of craniofacial surgery. 2012;23(1):338–42. Epub 2012/02/18. 10.1097/SCS.0b013e318241dbc4 . 22337438

[21] Kapp-Simon KA, Wallace E, Collett BR, Cradock MM, Crerand CE, Speltz ML. Language, learning, and memory in children with and without single-suture craniosynostosis. J Neurosurg Pediatr. 2016:1–11. 10.3171/2015.9.PEDS15238 . 26722698

[22] Twigg SR, Wilkie AO. New insights into craniofacial malformations. Human molecular genetics. 2015 10.1093/hmg/ddv228 . 26085576 PMC4571997

[23] Muenke M, Kress W, Collmann H, Solomon B. Craniosynostoses: molecular genetics, principles of diagnosis and treatment Basel: Karger; 2011 x, 249 pages p.

[24] Furuya Y, Edwards MS, Alpers CE, Tress BM, Ousterhout DK, Norman D. Computerized tomography of cranial sutures. Part 1: Comparison of suture anatomy in children and adults. Journal of neurosurgery. 1984;61(1):53–8. Epub 1984/07/01. 10.3171/jns.1984.61.1.0053 . 6726411

[25] Merrill AE, Bochukova EG, Brugger SM, Ishii M, Pilz DT, Wall SA, et al Cell mixing at a neural crest-mesoderm boundary and deficient ephrin-Eph signaling in the pathogenesis of craniosynostosis. Human molecular genetics. 2006;15(8):1319–28. Epub 2006/03/17. 10.1093/hmg/ddl052 . 16540516

[26] Iseki S, Wilkie AO, Morriss-Kay GM. Fgfr1 and Fgfr2 have distinct differentiation- and proliferation-related roles in the developing mouse skull vault. Development. 1999;126(24):5611–20. Epub 1999/11/26. . 10572038 10.1242/dev.126.24.5611

[27] Clendenning DE, Mortlock DP. The BMP ligand Gdf6 prevents differentiation of coronal suture mesenchyme in early cranial development. PloS one. 2012;7(5):e36789 Epub 2012/06/14. 10.1371/journal.pone.0036789 22693558 PMC3365063

[28] Rice DP, Rice R, Thesleff I. Molecular mechanisms in calvarial bone and suture development, and their relation to craniosynostosis. European journal of orthodontics. 2003;25(2):139–48. Epub 2003/05/10. . 12737212 10.1093/ejo/25.2.139

[29] Walker MB, Kimmel CB. A two-color acid-free cartilage and bone stain for zebrafish larvae. Biotechnic & histochemistry: official publication of the Biological Stain Commission. 2007;82(1):23–8. Epub 2007/05/19. 10.1080/10520290701333558 . 17510811

[30] Parichy DM, Elizondo MR, Mills MG, Gordon TN, Engeszer RE. Normal table of postembryonic zebrafish development: staging by externally visible anatomy of the living fish. Developmental dynamics. 2009;238(12):2975–3015. Epub 2009/11/06. 10.1002/dvdy.22113 19891001 PMC3030279

[31] Kimmel CB, DeLaurier A, Ullmann B, Dowd J, McFadden M. Modes of developmental outgrowth and shaping of a craniofacial bone in zebrafish. PloS one. 2010;5(3):e9475 Epub 2010/03/12. 10.1371/journal.pone.0009475 20221441 PMC2832765

[32] Sabaliauskas NA, Foutz CA, Mest JR, Budgeon LR, Sidor AT, Gershenson JA, et al High-throughput zebrafish histology. Methods. 2006;39(3):246–54. Epub 2006/07/28. 10.1016/j.ymeth.2006.03.001 . 16870470

[33] Filipek-Gorniok B, Holmborn K, Haitina T, Habicher J, Oliveira MB, Hellgren C, et al Expression of chondroitin/dermatan sulfate glycosyltransferases during early zebrafish development. Developmental dynamics. 2013;242(8):964–75. 10.1002/dvdy.23981 . 23703795

[34] Dolber PC, Spach MS. Conventional and confocal fluorescence microscopy of collagen fibers in the heart. The journal of histochemistry and cytochemistry. 1993;41(3):465–9. Epub 1993/03/01. . 7679127 10.1177/41.3.7679127

[35] Slater BJ, Liu KJ, Kwan MD, Quarto N, Longaker MT. Cranial osteogenesis and suture morphology in Xenopus laevis: a unique model system for studying craniofacial development. PloS one. 2009;4(1):e3914 Epub 2009/01/22. 10.1371/journal.pone.0003914 19156194 PMC2615207

[36] Jasinoski SC, Reddy BD, Louw KK, Chinsamy A. Mechanics of cranial sutures using the finite element method. Journal of biomechanics. 2010;43(16):3104–11. Epub 2010/09/10. 10.1016/j.jbiomech.2010.08.007 . 20825945

[37] Karsenty G. Minireview: transcriptional control of osteoblast differentiation. Endocrinology. 2001;142(7):2731–3. Epub 2001/06/21. 10.1210/endo.142.7.8306 . 11415989

[38] Viguet-Carrin S, Garnero P, Delmas PD. The role of collagen in bone strength. Osteoporosis international. 2006;17(3):319–36. Epub 2005/12/13. 10.1007/s00198-005-2035-9 . 16341622

[39] Wei J, Shimazu J, Makinistoglu MP, Maurizi A, Kajimura D, Zong H, et al Glucose Uptake and Runx2 Synergize to Orchestrate Osteoblast Differentiation and Bone Formation. Cell. 2015;161(7):1576–91. 10.1016/j.cell.2015.05.029 26091038 PMC4475280

[40] Komori T. Regulation of bone development and extracellular matrix protein genes by RUNX2. Cell Tissue Res. 2010;339(1):189–95. 10.1007/s00441-009-0832-8 . 19649655

[41] Eames BF, Amores A, Yan YL, Postlethwait JH. Evolution of the osteoblast: skeletogenesis in gar and zebrafish. BMC Evol Biol. 2012;12:27 10.1186/1471-2148-12-27 22390748 PMC3314580

[42] Avaron F, Hoffman L, Guay D, Akimenko MA. Characterization of two new zebrafish members of the hedgehog family: atypical expression of a zebrafish indian hedgehog gene in skeletal elements of both endochondral and dermal origins. Developmental dynamics. 2006;235(2):478–89. 10.1002/dvdy.20619 . 16292774

[43] Renn J, Buttner A, To TT, Chan SJ, Winkler C. A col10a1:nlGFP transgenic line displays putative osteoblast precursors at the medaka notochordal sheath prior to mineralization. Developmental biology. 2013;381(1):134–43. 10.1016/j.ydbio.2013.05.030 . 23769979

[44] Geurtzen K, Knopf F, Wehner D, Huitema LF, Schulte-Merker S, Weidinger G. Mature osteoblasts dedifferentiate in response to traumatic bone injury in the zebrafish fin and skull. Development. 2014;141(11):2225–34. 10.1242/dev.105817 . 24821985

[45] Knopf F, Hammond C, Chekuru A, Kurth T, Hans S, Weber CW, et al Bone regenerates via dedifferentiation of osteoblasts in the zebrafish fin. Developmental cell. 2011;20(5):713–24. 10.1016/j.devcel.2011.04.014 . 21571227

[46] DeLaurier A, Eames BF, Blanco-Sanchez B, Peng G, He X, Swartz ME, et al Zebrafish sp7:EGFP: a transgenic for studying otic vesicle formation, skeletogenesis, and bone regeneration. Genesis. 2010;48(8):505–11. Epub 2010/05/28. 10.1002/dvg.20639 20506187 PMC2926247

[47] Nakashima K, Zhou X, Kunkel G, Zhang Z, Deng JM, Behringer RR, et al The novel zinc finger-containing transcription factor osterix is required for osteoblast differentiation and bone formation. Cell. 2002;108(1):17–29. Epub 2002/01/17. . 11792318 10.1016/s0092-8674(01)00622-5

[48] Sinha KM, Zhou X. Genetic and molecular control of osterix in skeletal formation. Journal of cellular biochemistry. 2013;114(5):975–84. Epub 2012/12/12. 10.1002/jcb.24439 23225263 PMC3725781

[49] Bhat A, Boyadjiev SA, Senders CW, Leach JK. Differential growth factor adsorption to calvarial osteoblast-secreted extracellular matrices instructs osteoblastic behavior. PloS one. 2011;6(10):e25990 Epub 2011/10/15. 10.1371/journal.pone.0025990 21998741 PMC3187840

[50] Komori T. Regulation of skeletal development by the Runx family of transcription factors. Journal of cellular biochemistry. 2005;95(3):445–53. 10.1002/jcb.20420 . 15786491

[51] Li N, Felber K, Elks P, Croucher P, Roehl HH. Tracking gene expression during zebrafish osteoblast differentiation. Developmental dynamics. 2009;238(2):459–66. 10.1002/dvdy.21838 . 19161246

[52] McGee-Lawrence ME, Li X, Bledsoe KL, Wu H, Hawse JR, Subramaniam M, et al Runx2 protein represses Axin2 expression in osteoblasts and is required for craniosynostosis in Axin2-deficient mice. The Journal of biological chemistry. 2013;288(8):5291–302. Epub 2013/01/10. 10.1074/jbc.M112.414995 23300083 PMC3581413

[53] Yoshida T, Phylactou LA, Uney JB, Ishikawa I, Eto K, Iseki S. Twist is required for establishment of the mouse coronal suture. Journal of anatomy. 2005;206(5):437–44. 10.1111/j.1469-7580.2005.00411.x 15857364 PMC1571510

[54] Jacob AL, Smith C, Partanen J, Ornitz DM. Fibroblast growth factor receptor 1 signaling in the osteo-chondrogenic cell lineage regulates sequential steps of osteoblast maturation. Developmental biology. 2006;296(2):315–28. 10.1016/j.ydbio.2006.05.031 16815385 PMC2077084

[55] Sousa S, Valerio F, Jacinto A. A new zebrafish bone crush injury model. Biol Open. 2012;1(9):915–21. 10.1242/bio.2012877 23213486 PMC3507236

[56] Boskey AL, Maresca M, Ullrich W, Doty SB, Butler WT, Prince CW. Osteopontin-hydroxyapatite interactions in vitro: inhibition of hydroxyapatite formation and growth in a gelatin-gel. Bone Miner. 1993;22(2):147–59. . 8251766 10.1016/s0169-6009(08)80225-5

[57] Boskey AL, Spevak L, Paschalis E, Doty SB, McKee MD. Osteopontin deficiency increases mineral content and mineral crystallinity in mouse bone. Calcified tissue international. 2002;71(2):145–54. 10.1007/s00223-001-1121-z . 12073157

[58] Narisawa S, Yadav MC, Millan JL. In vivo overexpression of tissue-nonspecific alkaline phosphatase increases skeletal mineralization and affects the phosphorylation status of osteopontin. Journal of bone and mineral research. 2013;28(7):1587–98. 10.1002/jbmr.1901 23427088 PMC3688694

[59] Bieller A, Pasche B, Frank S, Glaser B, Kunz J, Witt K, et al Isolation and characterization of the human forkhead gene FOXQ1. DNA Cell Biol. 2001;20(9):555–61. 10.1089/104454901317094963 . 11747606

[60] Johnson D, Iseki S, Wilkie AO, Morriss-Kay GM. Expression patterns of Twist and Fgfr1, -2 and -3 in the developing mouse coronal suture suggest a key role for twist in suture initiation and biogenesis. Mechanisms of development. 2000;91(1–2):341–5. . 10704861 10.1016/s0925-4773(99)00278-6

[61] Hermann CD, Lee CS, Gadepalli S, Lawrence KA, Richards MA, Olivares-Navarrete R, et al Interrelationship of cranial suture fusion, basicranial development, and resynostosis following suturectomy in twist1(+/-) mice, a murine model of saethre-chotzen syndrome. Calcified tissue international. 2012;91(4):255–66. Epub 2012/08/21. 10.1007/s00223-012-9632-3 . 22903506

[62] Irie K, Zalzal S, Ozawa H, McKee MD, Nanci A. Morphological and immunocytochemical characterization of primary osteogenic cell cultures derived from fetal rat cranial tissue. The Anatomical record. 1998;252(4):554–67. Epub 1998/12/09. . 9845206 10.1002/(SICI)1097-0185(199812)252:4<554::AID-AR6>3.0.CO;2-2

[63] Moursi AM, Winnard PL, Winnard AV, Rubenstrunk JM, Mooney MP. Fibroblast growth factor 2 induces increased calvarial osteoblast proliferation and cranial suture fusion. The Cleft palate-craniofacial journal. 2002;39(5):487–96. Epub 2002/08/23. 10.1597/1545-1569(2002)039<0487:FGFIIC>2.0.CO;2 . 12190335

[64] Boonrungsiman S, Gentleman E, Carzaniga R, Evans ND, McComb DW, Porter AE, et al The role of intracellular calcium phosphate in osteoblast-mediated bone apatite formation. Proceedings of the National Academy of Sciences of the United States of America. 2012;109(35):14170–5. Epub 2012/08/11. 10.1073/pnas.1208916109 22879397 PMC3435222

[65] Spiesz EM, Kaminsky W, Zysset PK. A quantitative collagen fibers orientation assessment using birefringence measurements: calibration and application to human osteons. Journal of structural biology. 2011;176(3):302–6. Epub 2011/10/06. 10.1016/j.jsb.2011.09.009 21970947 PMC3218218

[66] Junqueira LC, Bignolas G, Brentani RR. Picrosirius staining plus polarization microscopy, a specific method for collagen detection in tissue sections. The Histochemical journal. 1979;11(4):447–55. Epub 1979/07/01. . 91593 10.1007/BF01002772

[67] Puchtler H, Waldrop FS, Valentine LS. Polarization microscopic studies of connective tissue stained with picro-sirius red FBA. Beitrage zur Pathologie. 1973;150(2):174–87. Epub 1973/11/01. . 4129194 10.1016/s0005-8165(73)80016-2

[68] Warren SM, Walder B, Dec W, Longaker MT, Ting K. Confocal laser scanning microscopic analysis of collagen scaffolding patterns in cranial sutures. The Journal of craniofacial surgery. 2008;19(1):198–203. Epub 2008/01/25. 10.1097/scs.0b013e31815c8a9a 18216689 PMC2705761

