# Supplementary figures and images for: The Morphogenesis of Cranial Sutures in Zebrafish
Source: PLoS One. 2016 Nov 9;11(11):e0165775. doi: 10.1371/journal.pone.0165775 (PMC5102434; doi:10.1371/journal.pone.0165775)

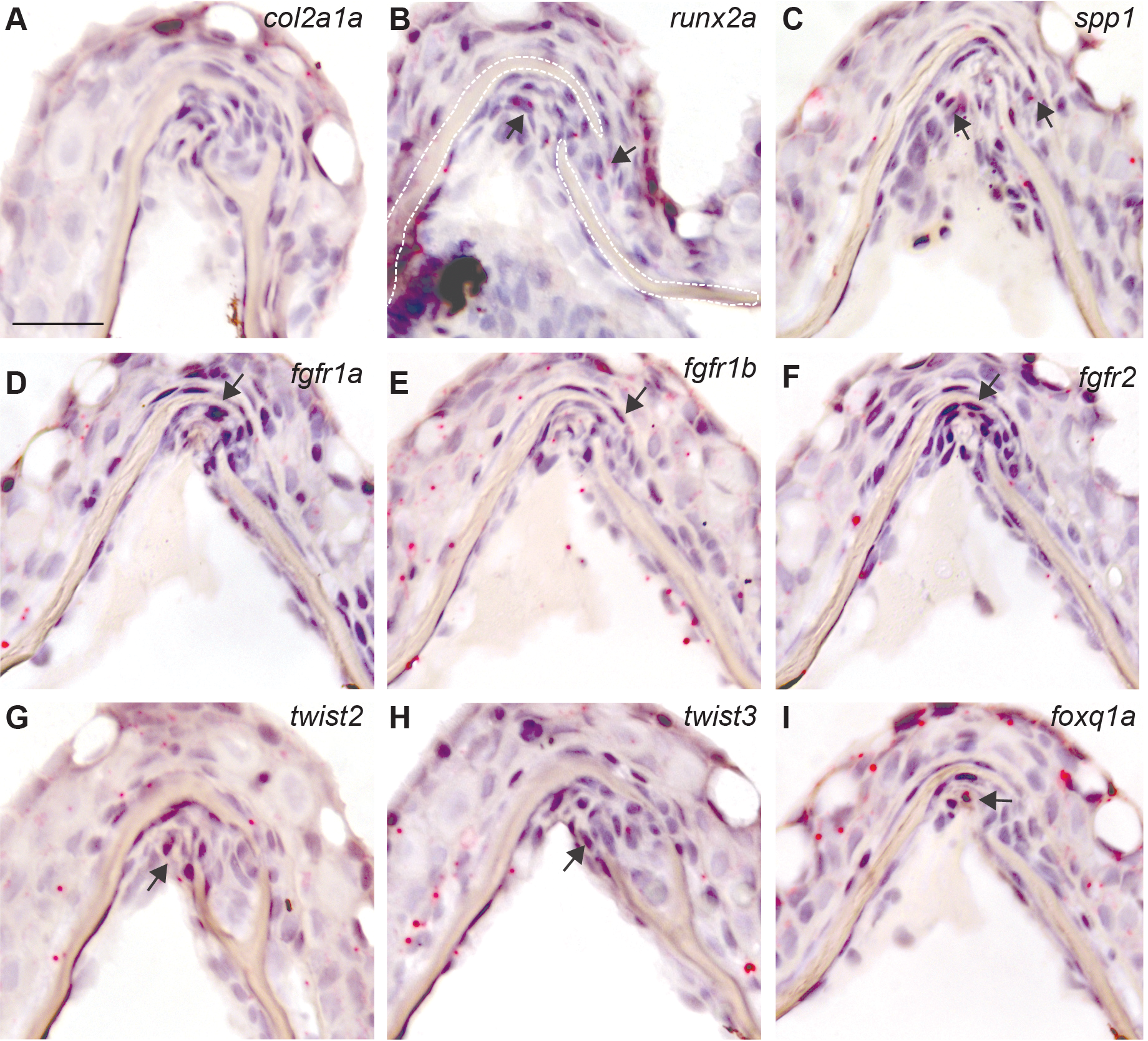

Supplement: S1 Fig — (A-I) Sequential sections (4 μm) of the interfrontal suture collected from juvenile zebrafish at age of 6 wpf. The expression of individual genes is visualized in red, counterstained with haematoxylin for nuclei in purple; black arrows indicate assumed positive expression. The expression of following genes is shown: (A) col2a1a, (B) runx2a, (C) spp1, (D) fgfr1a, (E) fgfr1b, (F) fgfr2, (G) twist2, (H) twist3, (I) foxq1a. The scale bar represents 20 μm. (TIF) [file pone.0165775.s001.tif]

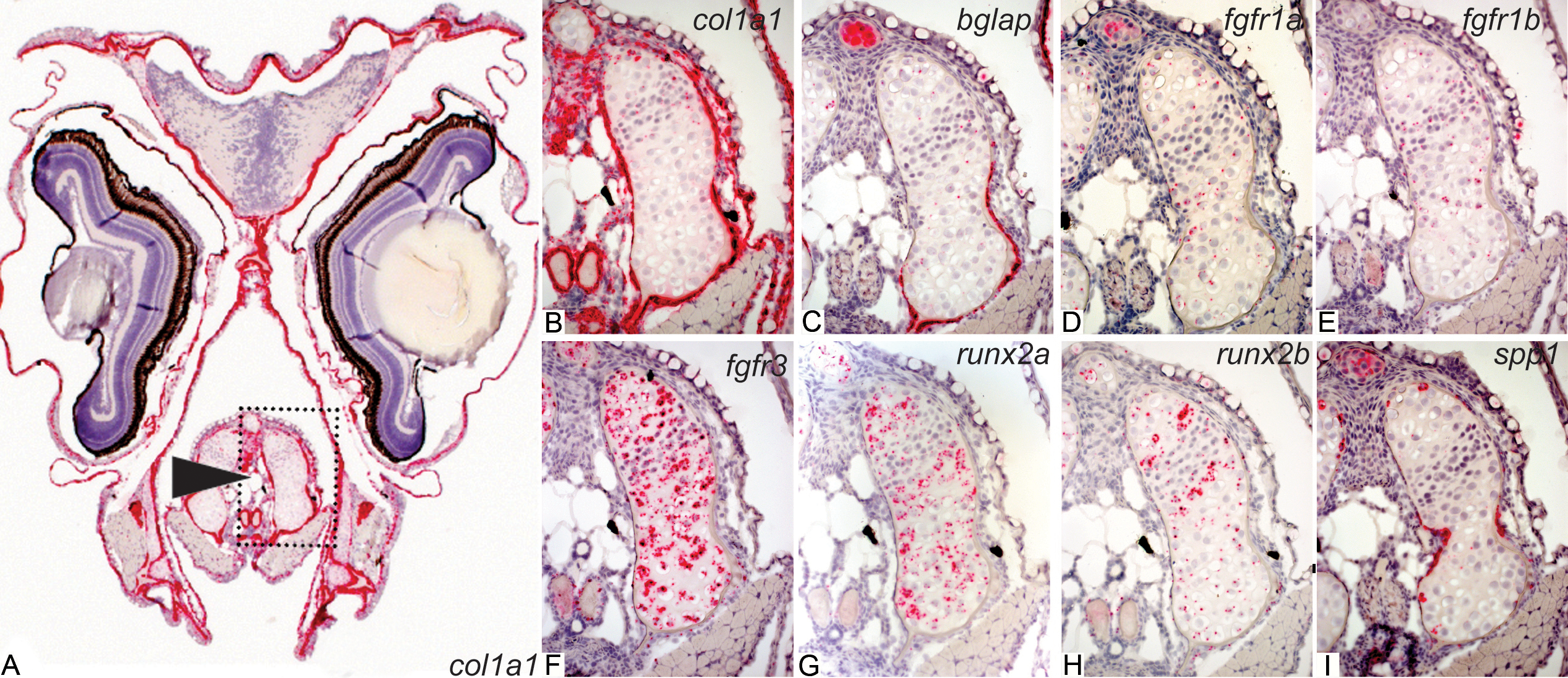

Supplement: S2 Fig — The images of the ceratohyal cartilage (dotted box and black arrowhead in A) collected from the tissue sections used for the interfrontal suture analysis shown in Fig 6 and S1 Fig. The expression of individual genes is visualized in red, counterstained with haematoxylin for nuclei in purple. The expression pattern of (A, B) col1a1a, (C) bglap, (D) fgfr1a, (E) fgfr1b, (F) fgfr3, (G) runx2a, (H) runx2b, (I) spp1. (TIF) [file pone.0165775.s002.tif]

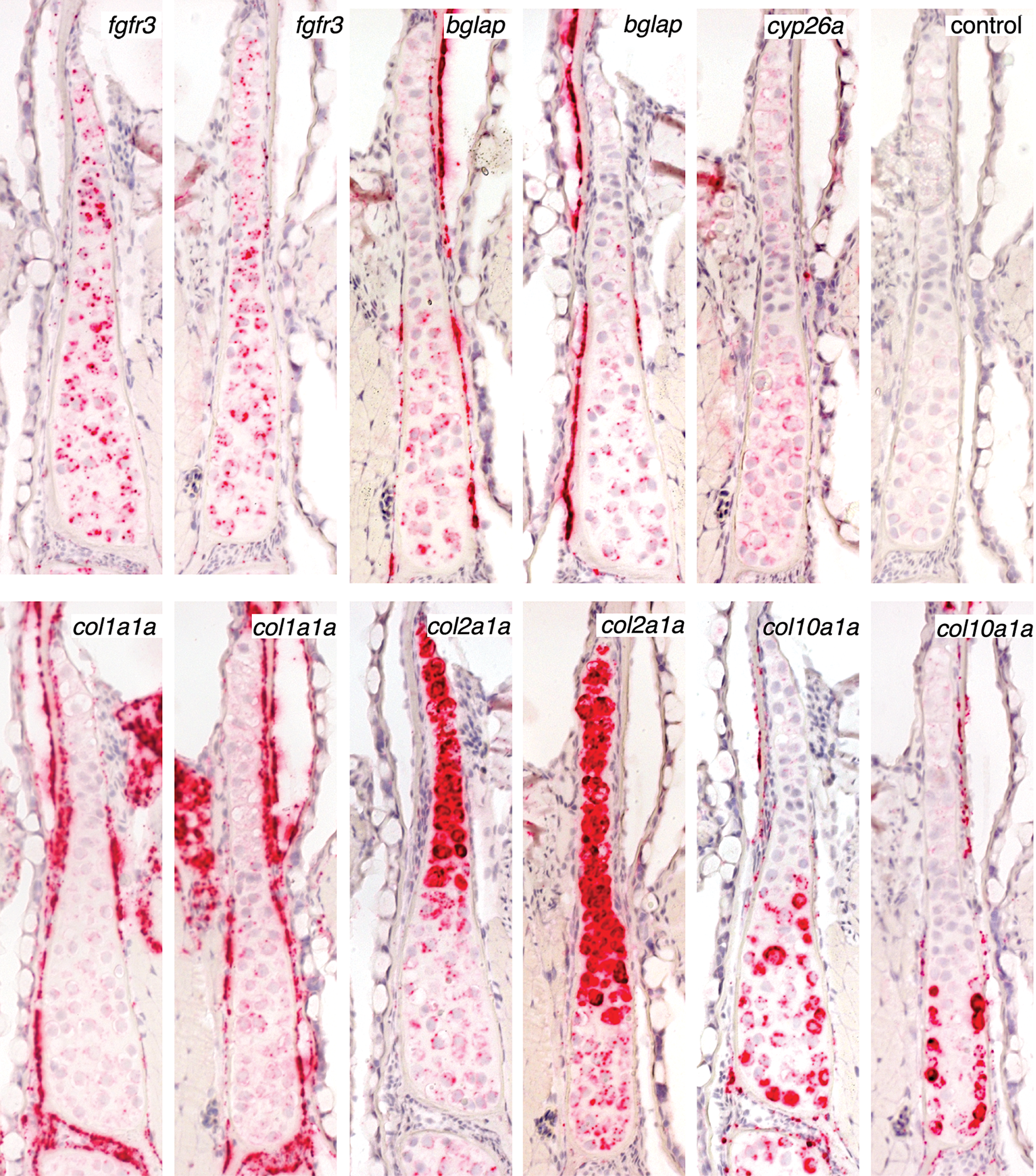

Supplement: S3 Fig — Both, the left and right palatoquadrate cartilages are shown as follows: fgfr3, bglap, cyp26a, col1a1a, col2a1a, col101a. The same tissue sections were analyzed for gene expression in the interfrontal suture as presented in Fig 6 and S1 and S2 Figs. (TIF) [file pone.0165775.s003.tif]
